# Synthesis, Radiolabelling and *In Vitro* Characterization of the Gallium-68-, Yttrium-90- and Lutetium-177-Labelled PSMA Ligand, CHX-A''-DTPA-DUPA-Pep

**DOI:** 10.3390/ph7050517

**Published:** 2014-04-29

**Authors:** Benjamin Baur, Christoph Solbach, Elena Andreolli, Gordon Winter, Hans-Jürgen Machulla, Sven N. Reske

**Affiliations:** 1 Clinic for Nuclear Medicine, University of Ulm, Albert-Einstein-Allee, 89081 Ulm, Germany; E-Mails: christoph.solbach@uniklinik-ulm.de (C.S.); elena.andreolli@gmail.com (E.A.); gordon.winter@uniklinik-ulm.de (G.W.); hans.machulla@uniklinik-ulm.de (H.-J.M.); sven.reske@uniklinik-ulm.de (S.N.R.); 2 Department of Health Sciences, University of Milano-Bicocca, Milan 20900, Italy

**Keywords:** PSMA, prostate-specific membrane antigen, PET, positron emission tomography, prostate cancer, DUPA, Ga-68, Y-90, Lu-177, radionuclide therapy

## Abstract

Since prostate-specific membrane antigen (PSMA) has been identified as a diagnostic target for prostate cancer, many urea-based small PSMA-targeting molecules were developed. First, the clinical application of these Ga-68 labelled compounds in positron emission tomography (PET) showed their diagnostic potential. Besides, the therapy of prostate cancer is a demanding field, and the use of radiometals with PSMA bearing ligands is a valid approach. In this work, we describe the synthesis of a new PSMA ligand, CHX-A''-DTPA-DUPA-Pep, the subsequent labelling with Ga-68, Lu-177 and Y-90 and the first *in vitro* characterization. In cell investigations with PSMA-positive LNCaP C4-2 cells, K_D_ values of ≤14.67 ± 1.95 nM were determined, indicating high biological activities towards PSMA. Radiosyntheses with Ga-68, Lu-177 and Y-90 were developed under mild reaction conditions (room temperature, moderate pH of 5.5 and 7.4, respectively) and resulted in nearly quantitative radiochemical yields within 5 min.

## 1. Introduction

Prostate cancer (PCa) is still one of the leading causes of cancer deaths among men. Despite using novel therapeutic approaches, mortality from metastasizing prostate cancer is still high [1]. Therefore, the early diagnosis of prostate cancer to prevent tumor dissemination is highly desirable. Furthermore, effective treatment strategies for disseminated prostate cancer are urgently needed. Comprehensibly, targeting of PCa or its metastases is a demanding task in the field of molecular imaging with positron emission tomography (PET) and for targeted internal radiation therapy.

Prostate-specific membrane antigen (PSMA) is a peptidase that catalyzes the hydrolysis of *N*-acetyl-l-aspartyl-l-glutamate (NAAG) into the corresponding *N*-acetyl-l-aspartate (NAA) and l-glutamate [2]. The use of PSMA as a target for diagnostic and therapeutic agents is a highly valid approach. Compared to healthy human prostate tissue, in almost all PCa tumors, the expression of PSMA is 10–80 fold higher.

Besides, the PSMA levels are increased in the neovasculature of other solid tumors, as well [3,4,5,6]. Therefore, selective addressing of PSMA with small molecules labelled with a positron emitting radionuclide is a considerable approach for the diagnosis of prostate cancer with PET.

Based on the chemical structure of NAAG, several glutamate-urea-glutamate-based peptides bearing a 2-[3-(1,3-dicarboxypropyl)-ureido]pentanedioic acid (DUPA) moiety were developed in the last few years [7]. These molecules showed high affinity and specific binding to PSMA, as demonstrated in binding studies using PSMA expressing LNCaP cell lines. Beside the slightly modified chemical structure, these molecules differ mainly in the selection of the chelator for the complexation of the desired radionuclide [7,8,9].

Due to their proven excellent affinity to PSMA, it is desirable to develop radionuclide-based therapeutic strategies using adapted PSMA targeting. In radiometal therapy approaches, the application of Y-90 and Lu-177 is favored. The use of Y-90 (E _βmax_: 2.3 MeV, t_½_: 64 h) is more appropriate in the treatment of larger tumor lesions, while Lu-177 (E_βmax_: 0.5 MeV, t_½_: 6.7 d) is more suitable for the treatment of smaller lesions and metastases, accompanied by a minimization of kidney dose in comparison to the application of Y-90 labelled peptides [10]. Moreover, due to the contemporary beta- and gamma-emission, Lu-177 is a useful diagnostic tool for scintigraphy of tumoral uptake [11].

For both diagnostic and therapeutic application, 1,4,7,10-tetraazacyclododecane-*N*,*N*',*N*'',*N*'''-tetraacetic acid (DOTA) is the mostly used chelator for the complexation of radiometals, like Ga-68, Lu-177 and Y-90, to small molecules [12,13]. However, DOTA can show some undesirable characteristics for therapy. Possible immunogenicity in humans has been published, as well as unfavorable kinetics in the complexation of radiometals [14,15]. Furthermore, labelling reactions using DOTA as the chelating agent are usually carried out at high temperatures under acidic conditions and long reaction times [16]. The preferred labelling procedure for peptides should consist of a simple, fast and quantitative labelling step at room temperature and neutral pH to avoid decomposition. Therefore, the use of alternative chelators is a demanding approach. In addition, the chelator must provide sufficient stability *in vivo*.

The preceding developments based on the chelator, diethylenetriaminepentaacetic acid (DTPA), lead to the promising cyclohexyl substituted analogue, cyclohexyl-diethylene triamine pentaacetic acid (CHX-A''-DTPA) [17]. It showed high stability *in vivo*, and radiolabelling with Y-90 was achieved under mild conditions (pH = 6 at room temperature) [17]. Thus, CHX-A''-DTPA is a highly promising alternative to the mostly used DOTA. Previous work using the PSMA ligand (5S,8S,22S,26S)-1-amino-5,8-dibenzyl-4,7,10,19,24-pentaoxo-3,6,9,18,23,25-hexaazaoctacosane-22,26,28-tricarboxylic acid (DUPA-Pep), conjugated with DOTA, revealed a high PSMA-affinity with a K_D_ of 21.6 ± 0.4 nM measured in a PSMA-positive LNCaP C4-2 cell assay [18]. Furthermore, the first clinical application of [^68^Ga]Ga-DOTA-DUPA-Pep showed the feasibility of *in vivo* targeting. [^68^Ga]Ga-HBED-PSMA showed promising clinical results for prostate cancer patients so far [19,20].

**Scheme 1:**
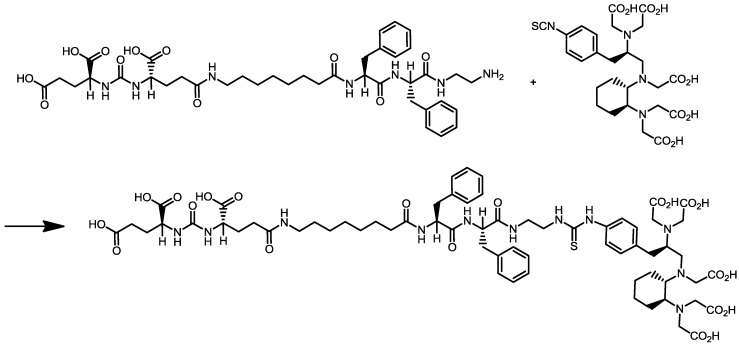
Synthesis of cyclohexyldiethylenetriamine pentaacetic acid (5*S*,8*S*,22*S*,26*S*)-1-amino-5,8-dibenzyl-4,7,10,19,24-pentaoxo-3,6,9,18,23,25-hexaazaoctacosane-22,26,28-tri-carboxylic acid trifluoroacetate (CHX-A''-DTPA-DUPA-Pep).

Thus, in the present study, we describe the synthesis of CHX-A''-DTPA-DUPA-Pep (Scheme 1), subsequent labelling with Ga-68, Y-90 and Lu-177, and the investigation of its biological activity as a new potential PSMA ligand for the diagnosis and therapy of prostate cancer. 

## 2. Experimental

[^90^Y]YCl_3_ and [^177^Lu]LuCl_4_, in 0.04 M HCl were purchased from Eckert and Ziegler and Isotope Technologies, respectively. The ^68^Ge/^68^Ga generator was obtained from IDB Holland BV. All chemicals and solvents were purchased from Aldrich, Fluka, Applichem, Waters and Merck and used without further purification. (5*S*,8*S*,22*S*,26*S*)-1-Amino-5,8-dibenzyl-4,7,10,19,24-pentaoxo-3,6,9,18,23,25-hexaazaoctacosane-22,26,28-tricarboxylic acid trifluoroacetate (“DUPA-Pep” trifluoroacetate) was obtained from ABX (Germany). *N*-[(*R*)-2-Amino-3-(*p*-isothiocyanato-phenyl)propyl]-trans-(*S*,*S*)-cyclohexane-1,2-diamine-*N*,*N*,*N*'-,*N*'',*N*''-pentaacetic acid (*p*-SCN-Bn-CHX-A''-DTPA) was achieved from Macrocyclics (Dallas, TX., USA). Diethylenetriaminepentaacetic acid (≥99%) was obtained from Fluka (Buchs, Switzerland). Solvents for analytics were of HPLC-grade purity. Chemicals for organic synthesis were of >98% purity. Chemicals for radiolabelling were of trace metal^®^ grade. An analytical assay was carried out by thin-layer chromatography (TLC) with Silica gel 60 RP-18 F_254_S (on alumina sheets of 5 × 7.5 cm, Merck) as the stationary phase, visualized by UV-detection for preparative synthesis and by phosphorimager analysis (FLA3000. Raytest, Straubenhardt, Germany) for radiosynthesis. In addition, a radio high-pressure liquid chromatography system (radio-HPLC, P680 Dionex, Idstein, Germany), equipped with NaI(TI)-scintillation detector (GABI, Raytest) and a UV-Vis detector (UVD 170U, Dionex), was used for the identification of the labelled peptide. For the control of radiochemical yields (RCYs), a γ-counter (Cobra^TM^ II, Packard Instrument, Canberra, Australia) was applied. ^1^H- and ^13^C- nuclear magnetic resonance spectra were recorded by a 400 MHz Bruker Avance spectrometer (Bruker, Karlsruhe, Germany) at ambient temperature. MS spectra were measured using a Triple Stage Quadrupole TSQ 70 Finnigan-MAT-mass spectrometer (Finnigan-MAT, Bremen, Germany).

### 2.1. Organic Synthesis of CHX-A''-DTPA-DUPA-Pep

DUPA-Pep trifluoroacetate (10 mg, 12.5 µmol) was dissolved in DMSO (1 mL), and DIPEA (15 µL, 90 µmol) and *p*-SCN-Bn-CHX-A''-DTPA·3HCl (Macrocyclics, Dallas, TX., USA) (7 mg, 11 nmol) were added. The reaction was carried out for 16 h. The product was purified by preparative HPLC consisting of a C18 column (Gemini 5µ C18, 250 × 21 mm, Phenomenex, Aschaffenburg, Germany) as the stationary phase and by use of gradient elution technique (gradient profile: 0–3 min 20% MeCN in H_2_O (0.1% TFA), 3–15 min from 20% to 40% MeCN in H_2_O (0.1% TFA), 15–30 min 40% MeCN in H_2_O (0.1% TFA); a flow rate of 7 mL/min, UV detection at 220 and 254 nm) as the mobile phase gave CHX-A''-DTPA-DUPA-Pep (12.5 mg, 8.6 µmol, 72%) as a colorless solid after lyophilization with a purity of >98% (HPLC). ^1^H NMR (400 MHz, DMSO-d_6_) δ = 1.2 (m, 14H); 1.7 (m, 4H); 2,0 (m, 8 H); 2.2 (m, 2H); 2.7 (m, 1H); 2.8 (m, 1H); 3.0 (m, 6H); 3.2 (m, 4H); 3.4 (m, 4H); 3.6 (m, 7H); 4.1 (m, 5H); 4.5 (m, 3H); 6.4 (m, 2H); 7.3 (m, 14H); 7.8 (m, 2H); 8.0 (m, 3H); 9.6 (s, 1H); 12.6 (s, 5H). ^13^C NMR (300 MHz, DMSO-d_6_) δ = 25.1 (2CH_2_); 26.3 (CH_2_); 27.5 (2*CH_2_); 28.3 (2*CH_2_); 28.3 (2*CH_2_); 28.4 (CH_2_); 28.5 (CH_2_); 29.1 (CH_2_); 29.8 (CH_2_); 31.6 (CH_2_); 35.1 (CH_2_); 37,0 (CH_2_); 37.2 (2*CH_2_); 37.7 (CH_2_); 37.9 (2*CH_2_); 38.5 (3*CH_2_); 48.3 (2*CH_2_); 51.6 (2*CH); 52.1 (CH); 53.6 (2*CH); 53.9 (2*CH); 126.1 (CH (phenylalanine)); 126.3 (CH (phenylalanine)); 127.9 (2*C2 phenyl(desferal)); 128.0 (2*C3 phenyl(desferal)); 129.1 (4*CH (phenylalanine)); 129.2 (4*CH (phenylalanine)); 137.5 (C1, C6 phenyl(desferal)); 137.9 (2*C (phenylalanine)); 155.3 (2*C = O); 157.2 (HN-(C = O)-NH); 158.2 (2*C = O); 171.0 (2*COOH); 171.1 (COOH); 172.1 (2*COOH); 173.7 (COOH); 174.1 (2*COOH); 174.2 (C = S). ESI-HRMS (*m*/*z*): MALDI calcd. for C_65_H_89_N_11_O_21_S, 1391,60; found, [M + H]^+^1392.60177.

### 2.2. Radionuclide Production and Radiochemistry

#### 2.2.1. Production of [^68^Ga]Ga-Chloride

Ga-68 (t_½_ = 67.7 min, β^+^: 89%, E_β_^+^_max_, 1.9 MeV; EC: 11%, E_γ max_: 4.0 MeV) was prepared as described by Meyer *et al.* [21]. Briefly, Ga-68 was eluted from a TiO_2_-based ^68^Ge/^68^Ga generator with *ca*. 2 mL of 1 M HCl and collected into a vial containing 1 mL of 9.5 M HCl. The resulting solution was loaded onto a strong anion exchange resin (100 mg Dowex 1 × 8), and the activity was retained as the [GaCl_4_]^−^ complex. After elution with 200 µL of water, the pH of the solution was neutralized by adding the appropriate volume of 2 M Na_2_CO_3_.

#### 2.2.2. Radiosynthesis of [^68^Ga]Ga-CHX-A''-DTPA-DUPA-Pep

The Ga-68 activity in neutral aqueous solution (13 µL; 9.3–9.6 MBq) was added to a freshly prepared solution of CHX-A''-DTPA-DUPA-Pep, dissolved in DMSO (2 mg/mL) and 0.25 M HEPES buffer (pH 7.4, 500 µL), resulting in a total volume of 514–563 µL and a final pH of 7.4. Different amounts of peptide (1, 10, 25, 50 and 100 µg; 0.7, 7.2, 18, 36 and 72 nM) were used to study the dependence of RCY on peptide concentration and reaction time. The reaction was carried out at room temperature for 60 min. At different reaction times (1, 5, 10, 30 and 60 min), 1-μL aliquots were withdrawn and analyzed by radio-TLC on RP-18 thin layer plates as the stationary phase and 0.1% TFA/MeOH 30/70 *v*/*v* as the mobile phase.

#### 2.2.3. Radiosynthesis of [^90^Y]Y-CHX-A''-DTPA-DUPA-Pep

Y-90 activity in 0.04 M HCl solution (20 µL; 3.7–3.8 MBq) was added to a freshly prepared solution of CHX-A''-DTPA-DUPA-Pep, dissolved in DMSO (2 mg/mL) and 0.5 M NH_4_OAc buffer (pH 5.5, 500 µL), resulting in a total volume of 533–570 µL and a final pH of 5.5. Different amounts of peptide (25, 50 and 100 µg; 18, 36 and 72 nM) were used to study the dependence of RCY on peptide concentration and reaction time. The reaction was carried out at room temperature for 60 min. At different reaction times (5, 10, 30 and 60 min), 1-μL aliquots were withdrawn and analyzed by radio-TLC on RP-18 thin layer plates as the stationary phase and 0.1% TFA/MeOH 30/70 *v*/*v* as the mobile phase.

#### 2.2.4. Radiosynthesis of [^177^Lu]Lu-CHX-A''-DTPA-DUPA-Pep

Lu-177 activity in 0.04 M HCl solution (5 µL; 6.3–6.7 MBq) was added to a freshly prepared solution of CHX-A''-DTPA-DUPA-Pep, dissolved in DMSO (2 mg/mL) and 0.5 M NH_4_OAc buffer (pH 5.5, 500 µL), resulting in to total volume of 510–555 µL and a final pH of 5.5. Different amounts of peptide (10, 25, 50 and 100 µg; 7.2, 18, 36 and 72 nM) were used to study the dependence of RCY on peptide concentration and reaction time. The reaction was carried out at room temperature for 60 min. At different reaction times (5, 10, 30 and 60 min), 1-μL aliquots were withdrawn and analyzed by radio-TLC on RP-18 thin layer plates as the stationary phase and 0.1% TFA/MeOH 30/70 *v*/*v* as the mobile phase.

#### 2.2.5. Radio-TLC Analysis

RCYs of the labelling reactions described above were determined in dependency on reaction time and peptide concentration. One microliter was withdrawn from every reaction mixture at different definite time points for radio-TLC analysis. RP-18 Silica gel plates were used as the stationary phase and 0.1% TFA/MeOH 30/70 *v*/*v* as the mobile phase, in which the free radiometal remains at the baseline (R_f_: 0.01). The R_f_ value of CHX-A''-DTPA-DUPA-Pep was found to be 0.73 (λ: 254 nm), while the R_f_ value of radio labelled compound was found to be *ca*. 0.78 (phosphorimager). Quantitative assays of radioactive spots were carried out by phosphorimager to determine the amount of radio labelled chelate and free radiometal.

#### 2.2.6. Radio-HPLC Analysis

**Figure 1:**
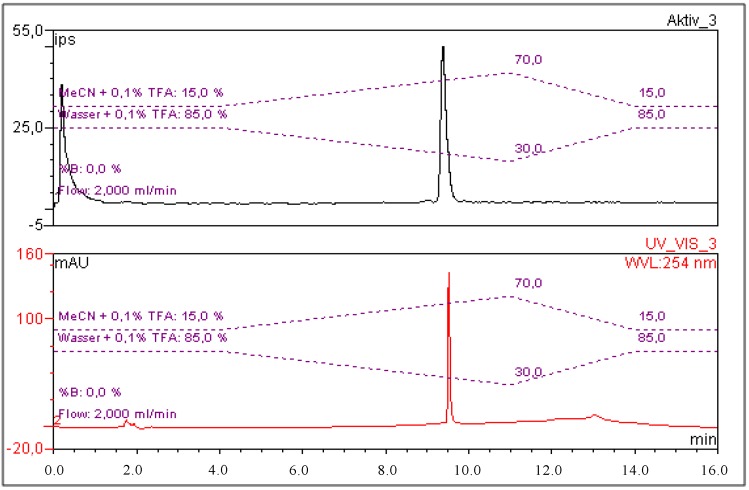
Exemplary chromatograms of [^68^Ga]Ga-CHX-A''-DTPA-DUPA-Pep in the radioactivity channel (upper signal; the first peak shows the total activity injected detector bypass for recovery rate calculation) and CHX-A''-DTPA-DUPA-Pep in the UV-channel (bottom signal; λ: 254 nm).

Radiochemical purity was determined by gradient radio-HPLC analysis. A C18 column (Phenomenex Gemini, 5 µm, C18, 250 × 4.6 mm) was used as stationary phase. Gradient elution technique was performed by the use of Solvent A (H_2_O, 0.1% TFA) and Solvent B (MeCN, 0.1% TFA) as the mobile phase (gradient profile: 85% A at 5 min; 85%–30% A from 5 to 14 min, 30%–85% A from 14 to 16 min; flow rate: 2 mL/min). The retention time of [^68^Ga]Ga-CHX-A''-DTPA-DUPA-Pep was 9.4 min (Figure 1).

### 2.3. Stability of Labelled CHX-A''-DTPA-DUPA-Pep

The stability of the labelled peptide in human serum and phosphate buffered saline (PBS) was verified via radio-HPLC after 30 min, 2, 4 and 8 h for [^68^Ga]Ga-CHX-A''-DTPA-DUPA-Pep and by radio-TLC on RP-18 thin layer plates as the stationary phase and 0.1% TFA/MeOH 30/70 *v*/*v* as the mobile phase after 1 h, 3 h, 6 h, 24 h, 48 h and 72 h for [^177^Lu]Lu-CHX-A''-DTPA-DUPA-Pep and [^90^Y]Y-CHX-A''-DTPA-DUPA-Pep. The radiochemical stability of the labelled compounds was determined in serum and PBS buffer at 37 °C. The total volume was 1.2 mL (980 µL of serum or buffer and 220 µL of product solution). In stability tests, 12 MBq of Ga-68 and 4.5 MBq of each radionuclide, Lu-177 and Y-90, were applied. Two hundred microliters of the mixture were withdrawn at each time point, and the samples were analyzed by radio-HPLC. Radio-TLC analyses were performed without further reprocessing.

The PBS samples were injected into a radio-HPLC system without further work-up. Serum samples of the Y-90 and Lu-177 labelled compound were tested without further work-up by radio-TLC. Serum samples for radio-HPLC-investigations underwent a serum protein precipitation work-up procedure before injection. Briefly: an equal volume of EtOH (200 µL) was added to the samples. Subsequently, the samples were centrifuged for 10 min at 10,000 rpm. Ultrafiltration of the supernatant was performed using an Amicon ultra centrifugal filter (3 KDa NMWL, Millipore, Darmstadt, Germany) for 10 min at 10,000 rpm. The filtrate was analyzed by radio-HPLC.

Exchange experiments with 500-fold excess of DTPA and additionally with hydrolysed *p*-SCN-Bn-CHX-A''-DTPA were performed for each of the three radiolabeled CHX-A''-DTPA-DUPA-Pep compounds. Briefly, after radiolabelling with Ga-68, Y-90 and Lu-177, a 500-fold excess of DTPA or hydrolysed *p*-SCN-Bn-CHX-A''-DTPA (in 500 µL of 0.5 M NH_4_OAc buffer (pH 5.5)) was added to the sealed reaction mixture stored at RT. Aliquots were withdrawn at two time points (30 min and 240 min) and analyzed by radio-TLC procedure (RP-18 Silica gel plates as the stationary phase and 0.1% TFA/MeOH 30/70 *v*/*v* as the mobile phase).

### 2.4. Cell Culture and Analysis of Binding Specificity of CHX-A''-DTPA-DUPA-Pep

The PSMA-positive cell line, LNCaP C4-2, was obtained from ViroMed Laboratories (Minnetonka, MN, USA) and grown in T-media (Dulbecco’s Modified Eagle’s Medium (DMEM), high glucose, Gibco) and 20% Ham’s F-12 (Biochrom, Berlin, Germany) supplemented with 5% fetal bovine serum, 5 µg/mL insulin (Sigma, St. Louis, MO., USA), 13.65 pg/mL triiodothyronine (Sigma), 5 µg/mL apo-transferrin (Sigma), 0.244 µg/mL D-biotin (Sigma) and 25 µg/mL adenine (Sigma). As a negative control, PC-3 cells (DSMZ, ACC 465) were maintained in DMEM and 10% Ham’s F12 with 10% FBS, 1% penicillin/streptomycin and 2 mM glutamine. All cell lines were incubated at 37 °C under constant humidity and an atmosphere of 5% CO_2_.

To determine the binding coefficient (K_D_) of the radio-labelled peptide, 5 × 10^5^ cells/well were grown in coated 12-well plates in 1 mL of medium for 48 h. The cells were washed twice with PBS, and 900 µL fresh media were added. Radiolabelled peptide was added, resulting in final concentrations of 480, 240, 120, 60, 30, 15 and 7.5 nM. In parallel, the PSMA-inhibitor (2-PMPA) was applied in a final concentration of 30 µM to determine unspecific binding. All samples were prepared in triplicate. Following 60 min of incubation at 37 °C, cells were washed twice to remove unbound activity and afterwards lysed in 1 mL of 0.5 M NaOH. Activity was measured in a gamma counter (COBRA^TM^ II, Packard Instrument). Aliquots of the solution added to the cells were also measured for the calculation of the cellular uptake as %ID. Data were analyzed using GraphPad Prism 5.02 (one site, total and non-specific binding evaluation).

### 2.5. Statistical Aspects

All experiments were conducted with *n* ≥ 3. Data are expressed as the mean ± SD.

## 3. Results and Discussion

### 3.1. Organic Synthesis of CHX-A''-DTPA-DUPA-Pep

CHX-A''-DTPA-DUPA-Pep synthesis was successfully achieved by coupling DUPA-Pep and p-SCN-Bn-CHX-A''-DTPA in the presence of DIPEA as the base. After preparative HPLC purification and lyophilization of the purified fraction, the peptide was obtained as a white powder in yields of 72% and a purity of ≥98% (HPLC).

### 3.2. Radiochemistry

**Figure 2:**
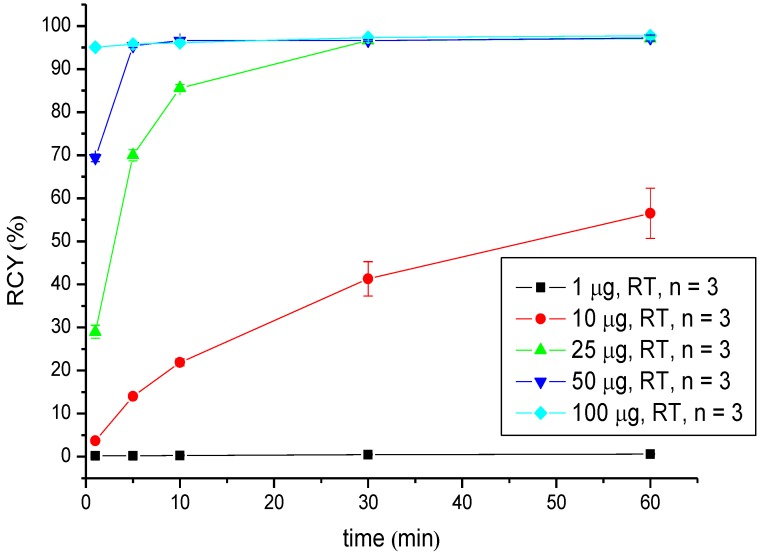
Dependence of the RCY of [^68^Ga]Ga-CHX-A''-DTPA-DUPA-Pep on the peptide amount (concentration) and reaction time at room temperature.

Radiolabelling with Ga-68 was performed in HEPES buffer (pH = 7.4) at room temperature by adding the prepared solution of [^68^Ga]GaCl_3_ to the peptide reaction mixture. The radiochemical yields were evaluated in dependence on the reaction time and the amount of peptide via radio-TLC (Table 1, Figure 2). Starting from 18 nM (25 µg) of CHX-A''-DTPA-DUPA-Pep, the RCY lay over 95% after 30 min of reaction time. Increasing the amount of peptide, to 72 nM (100 µg), a RCY >95% was obtained after 1 min of reaction time. Due to nearly quantitative labelling, the product solution was applied without further purification.

**Table 1:** Dependence of the radiochemical yield (RCY) of [^68^Ga]Ga-CHX-A''-DTPA-DUPA-Pep on the peptide amount (concentration) and the reaction time at room temperature.

Amount (µg)	RCY % (1 min)	RCY % (5 min)	RCY % (10 min)	RCY % (30 min)	RCY % (60 min)
1	0.2 ± 0 .1	0.2 ± 0.0	0.3 ± 0.1	0.5 ± 0.1	0.6 ± 0.1
10	3.7 ± 0.3	14.0 ± 0.3	21.9 ± 0.8	41.3 ± 4.0	56.5 ± 5.8
25	29.0 ± 1.5	70.0 ± 1.3	85.6 ± 0.8	96.6 ± 0.3	97.1 ± 0.6
50	69.4 ± 0.9	95.4 ± 0.4	96.6 ± 0.6	96.6 ± 0.2	97.2 ± 0.7
100	95.1 ± 0.3	95.8 ± 0.1	96.1 ± 0.1	97.3 ± 0.1	97.7 ± 0.6

**Figure 3:**
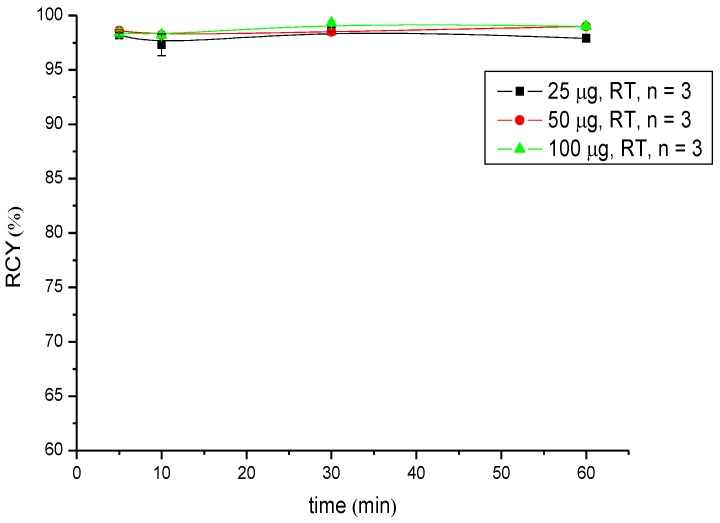
Dependence of the RCY of [^90^Y]Y-CHX-A''-DTPA-DUPA-Pep on peptide amount (concentration) and reaction time at room temperature.

**Figure 4:**
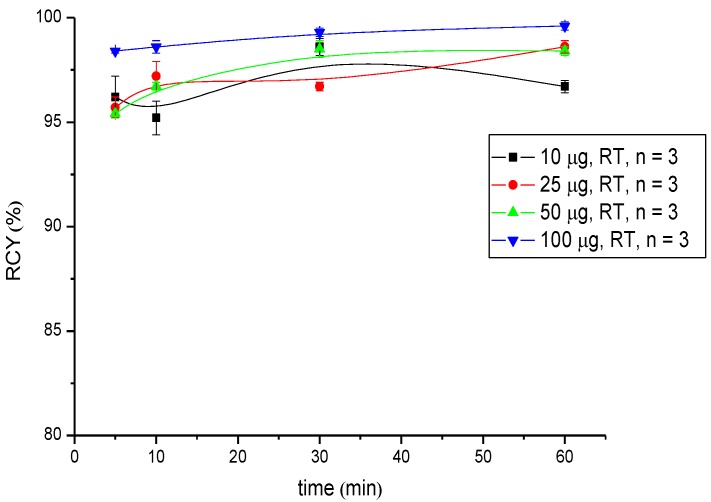
Dependence of the RCY of [^177^Lu]Lu-CHX-A''-DTPA-DUPA-Pep on peptide amount (concentration) and reaction time at room temperature.

Regarding radiometals for radiotherapy (Lu-177 and Y-90), the radiolabelling was performed at pH 5.5 in 0.5 M NH_4_OAc buffer and showed RCYs >95% for low amounts of peptide (25 µg (18 nM) in the case of ^9^^0^Y-labelling and 10 µg (7.2 nM) in the case of ^177^Lu-labelling) after 5 min of reaction time. A practically quantitative RCY of >99% was obtained in both radiosyntheses after 30 min of reaction time when the highest amount of peptide (100 µg; 72 nM) was used (Table 2 and Table 3, Figure 3 and Figure 4).

**Table 2:** Dependence of the RCY of [^90^Y]Y-CHX-A''-DTPA-DUPA-Pep on peptide amount (concentration) and reaction time at room temperature.

Amount (µg)	RCY % (5 min)	RCY % (10 min)	RCY % (30 min)	RCY % (60 min)
25	98.2 ± 0.2	97.3 ± 1.0	98.7 ± 0.2	97.9 ± 0.1
50	98.6 ± 0.1	98.2 ± 0.2	98.5 ± 0.3	99.0 ± 0.1
100	98.4 ± 0.4	98.2 ± 0.2	99.3 ± 0.2	99.0 ± 0.1

**Table 3:** Dependence of the RCY of [^177^Lu]Lu-CHX-A''-DTPA-DUPA-Pep on peptide amount (concentration) and reaction time at room temperature.

Amount (µg)	RCY % (5 min)	RCY % (10 min)	RCY % (30 min)	RCY % (60 min)
10	96.2 ± 1.0	95.2 ± 0.8	98.6 ± 0.4	96.7 ± 0.3
25	95.7 ± 0.5	97.2 ± 0.7	93.4 ± 0.4	96.1 ± 1.1
50	95.4 ± 0.1	96.7 ± 0.2	98.5 ± 0.4	98.4 ± 0.7
100	98.4 ± 0.1	98.6 ± 0.3	99.3 ± 0.2	99.6 ± 0.2

### 3.3. Stability Investigations

**Figure 5:**
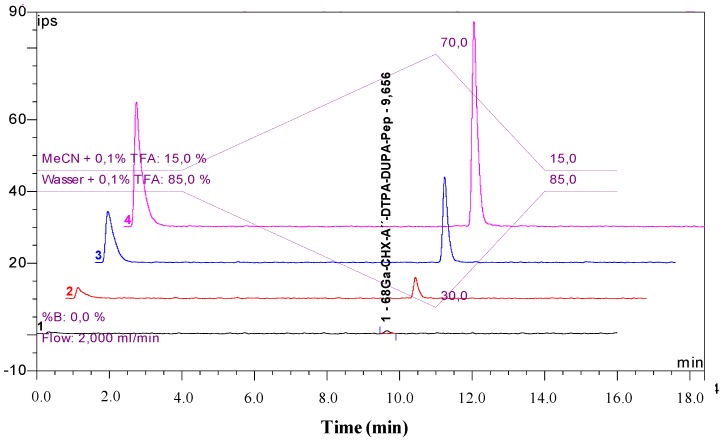
Stability study of [^68^Ga]Ga-CHX-A''-DTPA-DUPA-Pep in human serum: overlaid radio-HPLC chromatograms after 8 h (1), 4 h (2), 2 h (3) and 30 min (4). The first peak shows the total activity injected (the detector bypass for the recovery rate calculation).

A high incorporation level and high complex stability is strongly desired in therapeutic applications, due to the severe myelotoxicity associated with Y-90 and the long half-life of Lu-177 (6.74 d). Thus, in order to verify the stability of the Y-90 and Lu-177 complexes, stability studies were performed in human serum and PBS at 37 °C. Additionally, the stability of the Ga-68 labelled compound was investigated. Radio-TLC analysis of [^177^Lu]Lu- and [^90^Y]Y-CHX-A''-DTPA-DUPA-Pep after 1 h, 3 h, 6 h, 24 h, 48 h and 72 h confirmed the stability of the radiolabelled product. Moreover, the incubation of the [^68^Ga]Ga-CHX-A''-DTPA-DUPA-Pep for 30 min, 1 h, 2 h, 4 h and 8 h at 37 °C resulted in no detectable changes, as shown by radio-HPLC (Figure 5). Exchange experiments with a 500-fold excess of DTPA and with a 500-fold excess of hydrolysed *p*-SCN-Bn-CHX-A''-DTPA confirmed the stability of the investigated radiolabelled complexes. No radiometal labelled chelator (DTPA or hydrolysed *p*-SCN-Bn-CHX-A''-DTPA) was observed.

### 3.4. Determination of In Vitro Binding Specificity

The binding coefficient was investigated for all radiolabelled CHX-A''-DTPA-DUPA-Pep derivatives. In the PSMA-positive cell line, LNCaP C4-2, K_D_ values of 14.67 ± 1.95 nM for ^177^Lu-labelled peptide, 8.0 ± 1.1 nM for Y-90 labelled peptide and 12.09 ± 0.66 nM for ^68^Ga-labelled CHX-A''-DTPA-DUPA-Pep were determined. No specific binding could be observed for the PSMA-negative cell line, PC-3.

## 4. Conclusions

The synthesis of the DTPA-derivative bearing the PSMA targeting ligand, CHX-A''-DTPA-DUPA-Pep, was straightforward and efficient.

The radiolabelling of the new PSMA ligand with diverse radiometals, for both diagnosis and therapy, was investigated, and optimized conditions were developed.

Labelling with Ga-68 was performed at room temperature under neutral conditions. Significant differences in RCY were observed. Radio labelling with Ga-68 succeeds in a short reaction time with high radiochemical yields; ≥95% when 50 µg (36 nM) of the peptide was used. Additionally, the labelling of CHX-A''-DTPA-DUPA-Pep with Y-90 and Lu-177 was successfully developed. In both cases, the RCY was >95% after 5 min at room temperature using 25 µg (18 nM) peptide Y-90 and 10 µg (7.2 nM) Lu-177, respectively. In conclusion, RCYs over 95% up to quantitative yields were obtained for each radionuclide at room temperature at a moderate pH value.

In cell uptake experiments with the PSMA-positive cell line, LNCaP C4-2, the biological activity of CHX-A''-DTPA-DUPA-Pep was tested exemplarily for the Ga-68- and Lu-177- radiolabelled peptide. The obtained K_D_ values of 14.67 ± 1.95 nM for the Ga-68 labelled peptide, 8.0 ± 1.1 nM for the Y-90 labelled peptide and 12.09 ± 0.66 nM for the Lu-177 labelled peptide demonstrate high biological activity towards PSMA. The stability of the labelled peptide was confirmed in both human serum and PBS buffer. Additionally performed exchange experiments by use of DTPA and hydrolysed *p*-SCN-Bn-CHX-A''-DTPA confirmed the complex stability. The application of CHX-A''-DTPA as a chelator allows the choice of the suited radionuclide with respect to the selected (clinical) application. In the next step, it has to be elucidated if Ga-68, Y-90 and Lu-177 labelled CHX-A''-DTPA-DUPA-Pep may be useful ligands for the diagnosis and therapy of prostate cancer.
